# A brief review of Lynch syndrome: understanding the dual cancer risk between endometrial and colorectal cancer

**DOI:** 10.3389/or.2025.1549416

**Published:** 2025-05-16

**Authors:** Sneha Pallatt, Sibin Nambidi, Subhamay Adhikary, Antara Banerjee, Surajit Pathak, Asim K. Duttaroy

**Affiliations:** ^1^ Medical Biotechnology Lab, Faculty of Allied Health Sciences, Chettinad Academy of Research and Education (CARE), Chettinad Hospital and Research Institute (CHRI), Chennai, India; ^2^ Department of Nutrition, Institute of Basic Medical Sciences, Faculty of Medicine, University of Oslo, Oslo, Norway

**Keywords:** endometrial cancer, colorectal cancer, Lynch syndrome, genetic mutations, risk factors

## Abstract

Lynch syndrome (LS) is an autosomal dominant disorder caused by germline mutations in DNA mismatch repair (MMR) genes. These mutations result in frameshift alterations, leading to the accumulation of errors within microsatellites. Individuals with LS have an elevated risk of developing colorectal and distant malignancies, including endometrial cancer (EC), which is one of the most common cancer associated with LS. Despite its significance, the association between EC and LS is often underexplored. Given the slow progression of colorectal cancer (CRC), there is an opportunity for early detection and intervention, which can aid in reducing both incidence and mortality through the identification and management of pre-malignant lesions and early-stage tumors in colorectum/endometrium. Recognizing individuals with a heightened risk of CRC is essential for implementing personalized screening strategies. This review summarizes the original research work on LS to find out the correlation of CRC following an endometrial cancer diagnosis in individuals with MMR gene mutations, may involve refine treatment strategies and moreover this review may help clinicians and researchers to get an up-to date information on LS and its advanced treatment possibilities.

## Highlights

• This review comprehensively summarizes the current research findings on LS and possible correlation between CRC development following EC in individuals with MMR gene mutations.
• This review discussed the genetic and molecular pathways, such as MMR gene mutations and microsatellite instability (MSI), that drive the development of both EC and CRC.
• This review finds the key points regarding the role of early detection and surveillance strategies in LS carriers from the original research data available.

## 1 Overview of Lynch syndrome and associated cancer risks

Lynch syndrome (LS) is a hereditary condition that predisposes individuals to various malignancies, most notably colorectal cancer (CRC) and endometrial cancer (EC) (1). This autosomal dominant disorder is characterized by an increased cancer risk due to defects in DNA mismatch repair (MMR), which compromises genomic stability (2). Microsatellite instability (MSI) is a crucial screening factor for Lynch-associated tumors and underscores the aggressive and rapid progression of these cancers compared to sporadic cases (3, 4). A tumor is classified as microsatellite instability-high (MSI-H) when mutations are detected in two or more of the five microsatellite sequences within the tumor DNA. If only one of these five sequences is altered, the tumor is categorized as microsatellite instability-low (MSI-L). When none of the microsatellite sequences exhibit mutations, the tumor is considered microsatellite stable (MSS) (5). In cases where a tumor is identified as MSI-L, further testing with an extended panel of microsatellite markers is recommended to ensure precise classification (6). In LS, MSI-H tumors are primarily caused by germline mutations, while somatic mutations in the *MLH1* and *MSH2* genes are observed in only a small percentage of sporadic cases (7). The most common explanation for MSI-H tumors in sporadic cases is the silencing of the *MLH1* gene by promoter hyper-methylation, a phenomenon also observed in LS. Additionally, MSI-H tumors are strongly associated with the loss of *MLH1* protein expression in sporadic tumors, whereas familial tumors often exhibit a loss of both *MLH1* and *MSH2* protein expression (8). These genetic alterations create genomic instability, thereby expediting the progression of CRC in patients with LS, frequently advancing from adenoma to carcinoma in an approximate timeframe of 2 years, in stark contrast to the decade-long evolution observed in sporadic cases (9). Beyond LS, additional hereditary syndromes, exemplified by Cowden syndrome, which is marked by mutations in phosphatase and tensin homolog (*PTEN*), further enhance the risk of developing EC. Lifestyle determinants, such as obesity, physical inactivity, and specific dietary habits, exacerbate the likelihood of both EC and CRC, underscoring the necessity for comprehensive preventive measures (10–12).

A thorough comprehension of the interrelated risks associated with EC and CRC in LS is essential for the enhancement of early detection and therapeutic management. The identification of common genetic mutations and molecular pathways not only augments diagnostic accuracy but also facilitates the development of targeted therapeutic interventions that are efficacious against both forms of cancer. Understanding the genetic and molecular factors underlying this syndrome is crucial for early detection and effective management of affected individuals. This review seeks to elucidate these interconnections, with the objective of informing clinical guidelines and improving prognostic outcomes for individuals afflicted with LS.

## 2 LS: mechanism and impact

**FIGURE 1:**
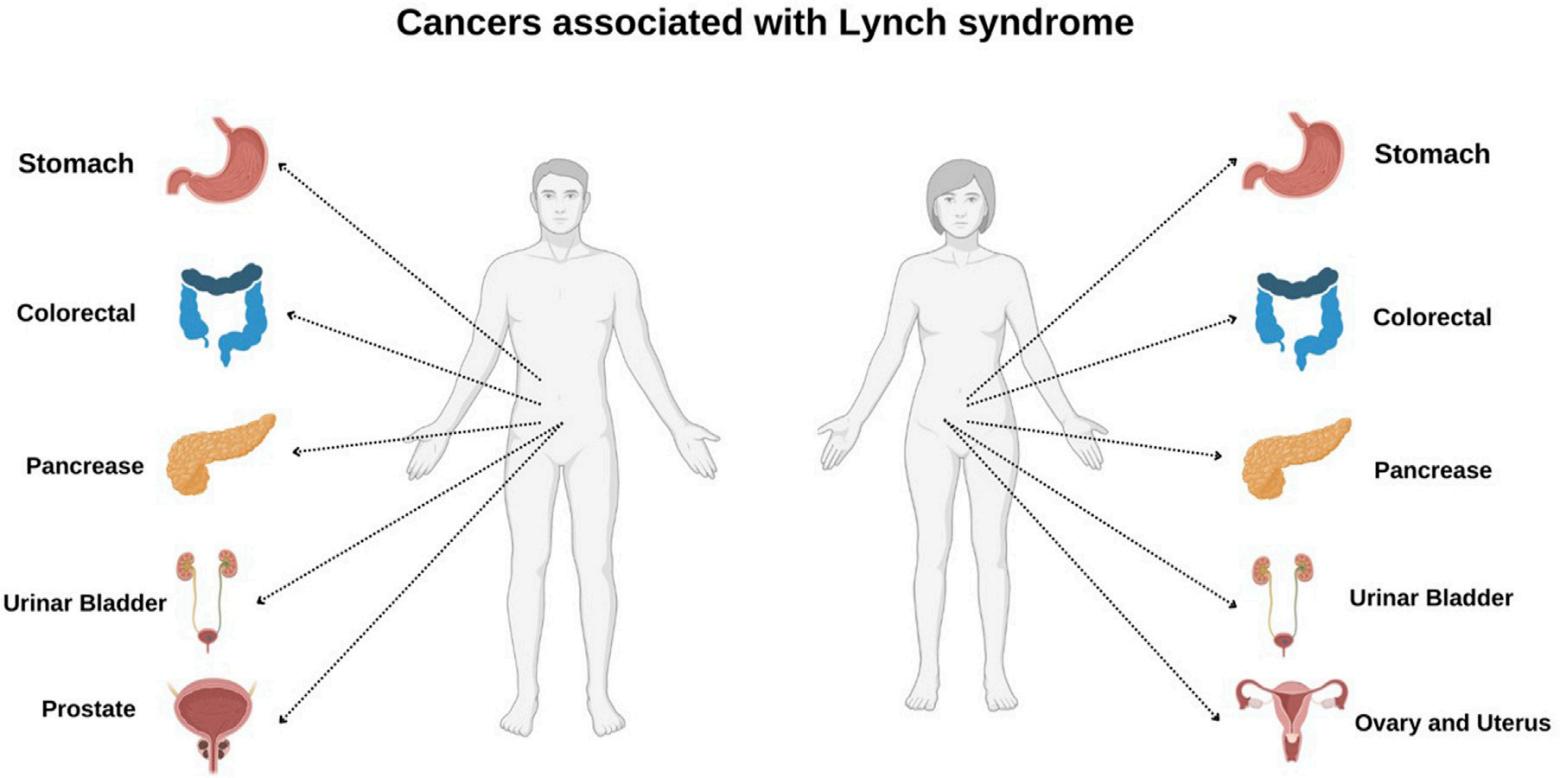
Cancer associated with Lynch syndrome in male and female.

Two major criteria are followed to classify individuals with LS, namely, Amsterdam Criteria II and Revised Bethesda Criteria mutations. The Amsterdam II criteria serve as a guideline for identifying families at high risk for LS, an autosomal dominant disorder that increases susceptibility to cancer. According to these criteria, a family must have at least three members diagnosed with cancers associated with LS, with at least one being a first-degree relative of the other two. Additionally, the disease should affect at least two successive generations, and at least one of the diagnosed individuals may have developed cancer before the age of 50. A confirmed pathological examination is required to verify the presence of tumors, and familial adenomatous polyposis must be ruled out as a possible cause (13). Similarly, Revised Bethesda Criteria is designed to recognize individuals with CRC who may require further evaluation for MSI and serve as a screening tool for LS. These guidelines assist in determining whether a patient’s tumor may be linked to MMR gene mutations, thereby indicating the need for additional genetic testing. One of the key indicators is early-onset CRC, where patients diagnosed before the age of 50 years require additional assessment due to an increased likelihood of hereditary cancer predisposition. Another critical criterion is the presence of synchronous or metachronous LS-associated malignancies, which include cancers of the colorectum, endometrium, stomach, ovaries, small intestine, biliary tract, ureter, or renal pelvis, occurring either concurrently or at different time points, necessitating genetic screening (Figure 1). Additionally, tumors exhibiting MSI-H histopathological features, such as mucinous differentiation, signet-ring cells, Crohn’s-like lymphocytic infiltration, or tumor-infiltrating lymphocytes, particularly when diagnosed before 60 years of age, suggest potential underlying MMR gene mutations and warrant further molecular analysis. Furthermore, a family history of early-onset CRC or LS-associated cancers in a first-degree relative (parent, sibling, or child) diagnosed before 50 years of age serves as another significant criterion for genetic testing. Lastly, the occurrence of CRC or other LS-associated malignancies in at least two first- or second-degree relatives (including grandparents, aunts, uncles, nephews, nieces, or grandchildren) at any age provides further justification for comprehensive genetic evaluation to identify hereditary cancer risks (14, 15). MSI results in changes in the length of microsatellites—short repetitive DNA sequences—and contributes to genomic instability, which drives tumorigenesis by enabling mutations in key oncogenes and tumor-suppressor genes such as *TGF-βR2*, *BAX*, and *PTEN*. MSI-related mutations in *TGF-βR2* impair cell proliferation regulation, while alterations in *BAX* hinder apoptosis, fostering tumor growth (16, 17).

### 2.1 Cancer spectrum and associated risks based on MMR gene variants

Investigations delineate a significant convergence in the genetic and molecular frameworks that support both EC and CRC. Mutations within mismatch repair genes, including *MLH1, MSH2*, *MSH6, PMS1*, and *PMS2*, play a crucial role in the origin of both malignancies (18). The risk and spectrum of cancers in LS vary depending on which MMR gene harbors the pathogenic variant, with each conferring distinct cancer risks and characteristics.

#### 2.1.1 MutL homolog 1(*MLH1*) and MutL homolog 2 (*MLH2*)

Individuals with pathogenic variants in *MLH1* and *MSH2* have the highest lifetime risk of CRC and EC, estimated between 40% and 80% (19). These individuals are also predisposed to distant colonic malignancies, including gastric, ovarian, urinary tract, hepatobiliary, and small bowel cancers. Among these, stomach cancer risk is particularly high in *MLH1* mutation carriers, with *MSH2* mutation carriers exhibiting a relatively lower but still significant risk (20). The variation in stomach cancer incidence between *MLH1* and *MSH2* carriers may be attributed to age-specific hazard ratio (HR) differences, a younger onset for *MLH1* carriers, or a higher representation of *MLH1* mutations among gastric cancer cases (21). Additionally, there is an increasing evidence for higher incidences of pancreatic cancer in LS carriers, as well as potential associations with breast and prostate cancers, given their frequent presentation with MMR deficiency in Lynch families (22). Moreover, a risk of cervical cancer has been noted, though some cases may be misclassified adenocarcinomas of the lower uterine segment rather than true cervical carcinomas. While the overall cumulative risks of LS-related cancers by age 70 are similar across *MLH1*, and *MSH2* mutation carriers, each mutated gene confers a unique cancer risk profile (23).

#### 2.1.2 MutS homolog 6 (*MSH6*)

Carriers of pathogenic *MSH6* mutations exhibit a distinct cancer risk profile within LS. Recent studies estimate the lifetime CRC risk for *MSH6* mutation carriers to range between 10% and 44%, typically presenting at a later age compared to *MLH1* or *MSH2* mutation carriers. However, the risk of EC is significantly elevated, with lifetime risks between 16% and 49%, often exceeding the risk of CRC (24). Additionally, *MSH6* mutations are associated with an increased but variable risk of ovarian cancer (25). Emerging evidence also suggests a heightened susceptibility to breast cancer, indicating a two-fold increased risk among *MSH6* and *PMS2* carriers compared to the general population. Other malignancies, including urinary tract, stomach, and small intestine cancers, have also been linked to *MSH6* mutations, though they occur less frequently (26).

#### 2.1.3 PMS1 homolog 2 (*PMS2*)

A defective *PMS2* gene associated with LS substantially elevates the possibilities of developing specific cancers, particularly CRC and EC in comparison to the general population. However, pathogenic *PMS2* variants are associated with the lowest cancer risks among LS-related MMR gene mutations. Studies indicate that the lifetime risk of CRC in individuals with *PMS2* mutations ranges between 10% and 20%, significantly lower than that of MLH1, MSH2, and MSH6 mutation carriers (27). Additionally, EC risk in *PMS2* carriers is estimated to be between 12% and 15%, also lower than those associated with other MMR genes. The later onset of CRC, typically occurring after age 50, contributes to a less aggressive screening approach. Unlike carriers of *MLH1* or *MSH2* mutations, who require biennial colonoscopy starting at age 20–25, *PMS2* mutation carriers may begin screening at age 35–40, with colonoscopies recommended every 2–3 years instead of annually (28). Recent studies have also suggested that *PMS2* carriers may have a lower risk of extra-colonic malignancies, though upper gastrointestinal, ovarian, and urinary tract cancers have been reported at lower frequencies. Due to the reduced overall cancer risk, prophylactic surgeries, such as hysterectomy, are not routinely recommended for *PMS2* carriers unless there is a strong family history of EC. *PMS2*-deficient CRCs tend to exhibit more aggressive behavior and a worse prognosis compared to other MMR-deficient CRCs (29). This distinction is partly attributed to lower levels of intra-tumoral immune infiltration, suggesting that *PMS2*-deficient CRCs share more biological characteristics with sporadic MMR-proficient CRCs than with other LS-associated CRCs. While it was previously believed that carriers of germline pathogenic *PMS2* variants represented a small minority of LS patients, recent studies have challenged this assumption. New investigations indicate that pathogenic *PMS2* carriers have the highest population frequency among the four MMR genes, with an estimated prevalence of 1 in 714 individuals (30). Furthermore, studies utilizing IHC staining in CRCs from population-based cohorts have demonstrated that isolated *PMS2* loss of expression, indicative of pathogenic *PMS2* variants, is observed in 0.5%–1.5% of unselected CRCs. Among MSI CRCs, the fraction of isolated *PMS2* loss varies between 1% and 8%, with more than half of these tumors being linked to germline pathogenic *PMS2* variants. These findings underscore the importance of refining screening strategies and risk assessment for *PMS2*-deficient CRCs to improve early detection and patient management (31).

#### 2.1.4 Epithelial cell adhesion molecule (*EPCAM*)

The *EPCAM* gene is not an MMR gene, but deletions in *EPCAM* lead to *MSH2* inactivation due to promoter hypermethylation, resulting in a cancer risk profile similar to *MSH2* variants (32). Individuals with *EPCAM* deletions have an increased risk of CRC, with studies reporting a lifetime risk of approximately 75%, comparable to *MSH2* mutation carriers. Additionally, the risk of EC in female carriers is estimated to be around 30%, reinforcing the need for targeted surveillance. Unlike other LS-associated mutations, *EPCAM* deletions do not directly affect DNA mismatch repair function but cause epigenetic silencing of *MSH2*, leading to a deficiency in MMR and MSI-H (33). This makes individuals with *EPCAM* deletions susceptible to other LS-associated cancers, including ovarian, gastric, small bowel, and urinary tract malignancies. Colonoscopy screening every 1–2 years starting at age 25 is recommended for *EPCAM* carriers, along with EC surveillance. However, because *EPCAM* deletions predominantly affect *MSH2* expression, further research is needed to refine cancer risk estimates and optimize screening protocols for affected individuals (34).

The autosomal dominant inheritance of LS results in a 50% probability of passing the condition to offspring, making genetic testing and counseling essential for at-risk families. Early and regular surveillance, such as colonoscopy starting at 20–25 years of age or 2–5 years before the youngest diagnosed family member, significantly reduces cancer-related mortality (35). Prophylactic surgical options, such as colectomy and hysterectomy, are also available for individuals at high risk. Importantly, tumors with MSI-H phenotypes in LS respond well to immune checkpoint inhibitors, particularly anti-PD-1/PD-L1 therapies, offering a targeted treatment approach (36). Advances in molecular diagnostics, including MSI testing and immunohistochemistry for MMR proteins, have greatly improved LS management, enabling timely interventions and personalized treatments to mitigate its impact on affected individuals and their families (37).

## 3 Endometrial cancer: a central player in LS’s cancer spectrum

EC represents a quintessential neoplasm within LS, frequently manifesting as the chief malignancy preceding the emergence of other tumors associated with LS, including CRC. It is estimated that approximately 40%–60% of female individuals with LS will experience the development of EC during their lifetimes, with the mean age of onset occurring 10–15 years earlier than that observed in sporadic, non-syndromic instances (38). The presence of MSI and germline mutations in MMR genes, particularly in *MSH2* and *MSH6*, is markedly prevalent in Lynch-associated EC, which contributes to genomic instability and tumorigenesis (39). In contrast to sporadic EC, which often relies on estrogen for its progression, Lynch-associated EC is generally non-estrogen-dependent and displays unique molecular subtypes, predominantly categorized as high-grade endometrioid carcinomas. Moreover, Lynch-associated EC is distinguished by a hyper-mutated phenotype, resulting in a high frequency of mutations in genes such as *PTEN, KRAS*, and *PIK3CA* (40). Estrogen-dependent EC is linked to factors that elevate lifetime exposure to endogenous or exogenous estrogens. These factors include a higher body mass index (BMI), estrogen replacement therapy, estrogen-secreting tumors, chronic anovulation, tamoxifen therapy, early onset of menstruation, and delayed menopause, all of which contribute to endometrial proliferation stimulated by estrogen (41). In contrast, non-estrogen-dependent EC is not associated with unopposed estrogen exposure and is linked to risk factors such as lower BMI, nulliparity, a history of breast cancer, and being over 55 years old at the time of diagnosis (42).

## 4 Colorectal cancer: insights from the LS perspective

Although CRC predominantly targets individuals aged 50 and above, those diagnosed with LS experience a considerably elevated risk and are frequently identified at a younger age due to the hereditary predisposition associated with their condition. Approximately 80% of hereditary CRC cases, particularly those associated with LS, arise via the mutation or alternative pathway linked to these MMR gene alterations. This is in contrast to the suppressor or classic pathway, which is responsible for around 80% of sporadic CRC instances, often connected to mutations in genes such as *APC*, *p53*, and *KRAS* (43). CRC associated with LS usually involves activation of the WNT/β-catenin signaling pathway due to secondary mutations in *APC* or β-catenin (*CTNNB1*), further advancing tumorigenic processes (9).

In individuals diagnosed with LS, CRC typically initiates as an adenomatous polyp within the intestinal mucosa, with malignant progression occurring at a considerably accelerated rate compared to sporadic cases (44). The typical duration from adenoma to carcinoma in Lynch-associated CRC is roughly 2 years, whereas this timeline extends to approximately 10 years for sporadic cases (27, 28). Unlike sporadic CRC, which often occurs in the distal colon and rectum, LS-associated CRCs predominantly arise in the proximal (right-sided) colon, particularly in the cecum and ascending colon (45). These tumors frequently display mucinous differentiation or signet-ring cell morphology and are poorly differentiated or undifferentiated, highlighting their aggressive nature. A characteristic immune response, marked by peri-tumoral and intra-tumorally lymphoid aggregates, is commonly observed, suggesting active immune surveillance against tumor cells.

Additionally, an increased presence of intraepithelial lymphocytes further reinforces their immunogenic nature, indicating a potential for responsiveness to immunotherapy (46). Some LS-associated CRCs also exhibit serrated glandular architecture or medullary carcinoma-like features, which are relatively uncommon in sporadic cases. A defining aspect of LS-associated CRCs is their rapid progression, transitioning from adenomatous polyps to invasive carcinoma within approximately 2 years, in contrast to the decade-long progression seen in sporadic CRCs (47). The clinical manifestations of CRC in patients possessing LS encompass symptoms including abdominal discomfort, alterations in bowel patterns, weight reduction, nausea, and anemia. Distal tumors are more inclined to induce visible rectal hemorrhage, whereas proximal tumors may lead to occult blood in the feces. In light of the distinctive hereditary risk factors, patients with LS may also exhibit atypical signs of metastasis, such as lymphadenopathy (e.g., Virchow’s node) or hepatomegaly (48).

## 5 Epidemiological insights and risk factors for EC and CRC

The epidemiology and risk factors for EC and CRC highlight unique and overlapping elements contributing to their development and prevalence. EC primarily impacts women in the postmenopausal stage, with a higher occurrence noted in correlation with advancing age (49). Risk determinants for endometrial carcinoma are closely associated with hormonal dysregulation, notably conditions that lead to extended exposure to estrogen without the counterbalancing effects of progesterone. Obesity, polycystic ovary syndrome (PCOS), nulliparity, and late menopause are significant contributors, as they increase endogenous estrogen levels (50). Estrogen promotes the growth of endometrial cells, raising the risk of hyperplasia (abnormal cell growth) and ultimately leading to EC. Progesterone opposes this effect by balancing estrogen’s action. It induces differentiation in endometrial cells, inhibits proliferation, and facilitates the shedding of the endometrial lining as seen during menstruation (51). When exogenous estrogen is given, such as in hormone replacement therapy (HRT) for postmenopausal women, without the addition of progesterone (unopposed estrogen therapy), the endometrial lining undergoes continuous stimulation without progesterone’s regulatory effects. This prolonged exposure can result in endometrial hyperplasia and markedly heighten the risk of developing EC. Lifestyle factors, including diets high in saturated fats and a lack of physical activity, further amplify this risk (52).

CRC has both genetic and environmental factors playing crucial roles in its epidemiology (53). Lifestyle factors such as diet, physical activity, and smoking are important modifiable risk factors (54). Diets high in red and processed meats, low fiber intake, and excessive alcohol consumption are associated with increased CRC risk. Additionally, chronic conditions such as inflammatory bowel disease (IBD), including Crohn’s disease and ulcerative colitis, elevate the risk of CRC (55). Women diagnosed with LS exhibit a markedly elevated probability of developing EC as their initial malignancy, frequently preceding the occurrence of CRC. This hereditary association emphasizes the critical necessity for systematic screening and vigilant surveillance in individuals possessing a familial predisposition to these malignancies (56,57).

## 6 LS associated EC and CRC genes

**FIGURE 2:**
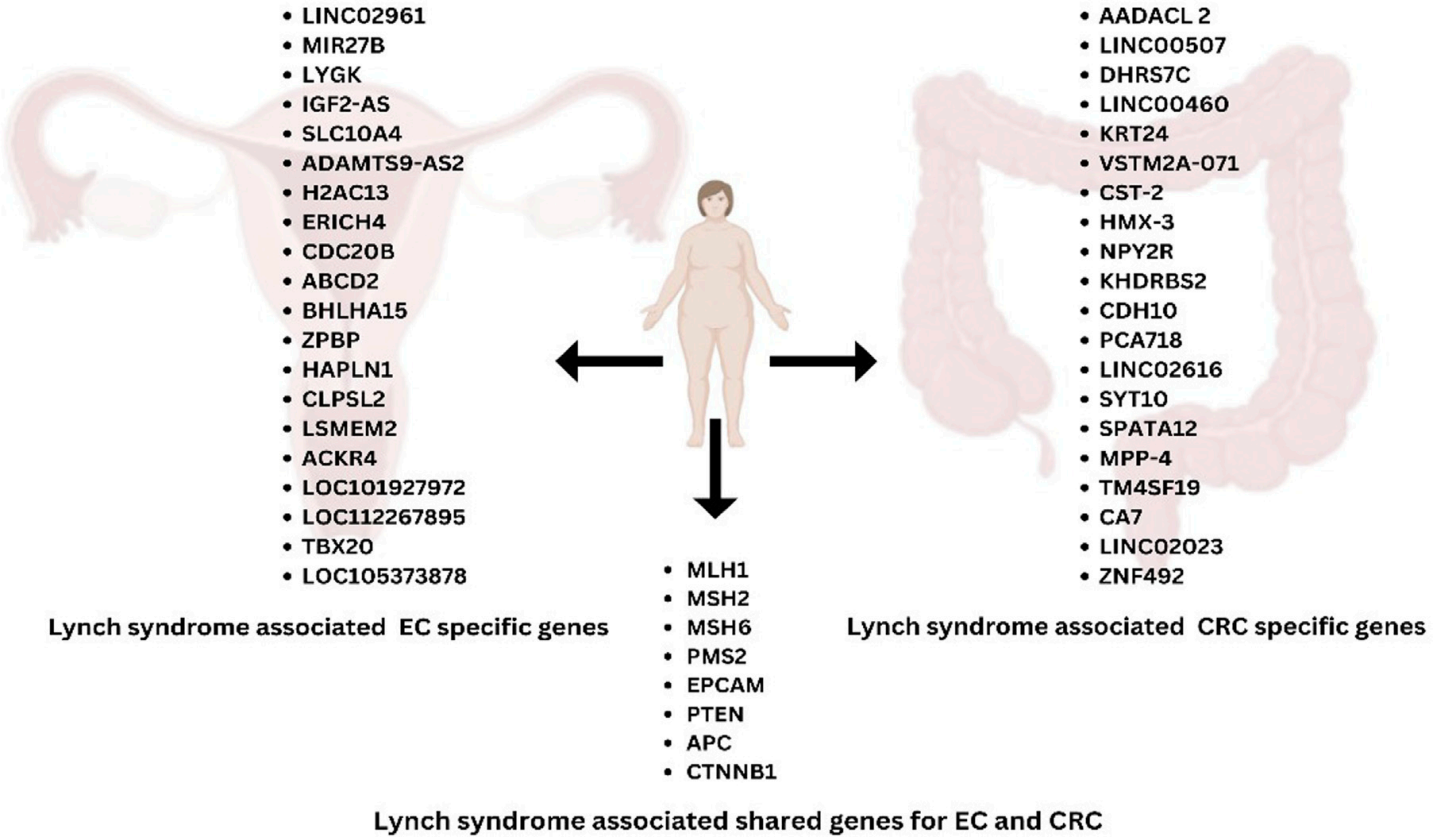
Lynch syndrome-associated genes specific to EC and CRC, highlighting the shared genes between EC and CRC.

A comprehensive analysis (Li et al., 2022) of data from the TCGA database revealed significant differences in the molecular mechanisms driving the progression of LS to CRC or EC. While LS-CRC progression is closely associated with differential gene expression (DEGs), LS-EC development may rely more on gene methylation processes. For instance, *COL11A1*, correlated with *MSH6* mutations, serves as a key marker for distinguishing MSI-H and microsatellite stable (MSS). CRC, playing a role in extracellular matrix interactions and tumor development (42). From the TCGA database, specific genes were identified that overlap with LS and CRC (SGs-LC) and LS and EC (SGs-LE), comprising 493 and 99 genes, respectively (Li et al., 2022). Enrichment analyses revealed distinct pathways for SGs-LC and SGs-LE, with shared associations in peroxisomal pathways but differing in other functional pathways. For SGs-LC, pathways related to peroxisomal activity and extracellular matrix remodeling may play pivotal roles, as evidenced by genes like *CST2* and *COL18A1* (58). In contrast, SGs-LE genes like *LY6K* and *MIR27B* are implicated in immune response modulation or hormone signaling, both critical in EC. Several genes exhibited notable roles in LS-associated tumor progression. SST, a regulatory peptide, inhibits cellular mitosis and tumor growth in various cancers, including CRC. Similarly, *KIF20A* and *NUF*2, implicated in mitotic regulation and tumorigenesis, show significant roles in both CRC and EC (58). Specific survival analyses further underscored unique and overlapping genetic markers influencing patient outcomes in CRC and EC. Genes like *COL18A1* and *HTR4* modulate the tumor microenvironment and signal transduction in CRC, while *CDC45* and *WDR31* influence cellular replication processes in EC. SGs-LC, genes such as *AADACL2, DHRS7C, KRT24*, and *LINC00460* exhibit highly significant p-values (59). Both upregulated (e.g., *LINC00460*) and downregulated (e.g., *AADACL2*) expressions have been noted, with *CST2* being significantly upregulated, suggesting its potential role in CRC tumor progression. Conversely, downregulated genes like *NPY2R* and *KHDRBS2* may contribute to CRC development through their suppression (59). Additional candidates, such as *CDH10* and *LINC02616*, are involved in CRC-specific pathways related to adhesion and cellular communication. For SGs-LE, genes like *LINC02691*, *MIR27B,* and *LY6K* are characterized by less pronounced but still significant differential expression. Notably, *IGF2-AS* is upregulated, potentially influencing the insulin-like growth factor (IGF) signaling pathway in EC. Meanwhile, genes like *ADAMTS9-AS2* and *SLC10A4* suggest potential epigenetic or regulatory functions in EC (Figure 2) (59).

## 7 Molecular alteration and dysregulation pathways in EC: distinction between endometrioid EC and serous EC

Endometrial endometrioid carcinomas (EECs) are marked by frequent genetic mutations and pathway dysregulations that drive their development and progression (60). EECs often exhibit MSI present in about 20% of unselected endometrial tumors and more common in EECs than non-EECs (61). This leads to mutations in various genes involved in tumorigenesis, including Birt-Hogg-Dube (*BHD*), *BAX*, insulin-like growth factor type 2 receptor (*IGFIIR*), Transforming Growth Factor-β Receptor II (*TGFβ-RII*), and ataxia telangiectasia and Rad3-related (ATR), many of which are part of the DNA damage response (62, 63). The PI3K-PTEN-AKT pathway is also significantly altered in over 80% of EECs, with high-frequency mutations in *PIK3R1*, *PIK3CA*, and *PTEN,* as well as additional alterations like *PIK3CA* amplification and *PTEN* promoter methylation. These mutations result in dysregulated cell proliferation, growth, and survival (64).

**FIGURE 3:**
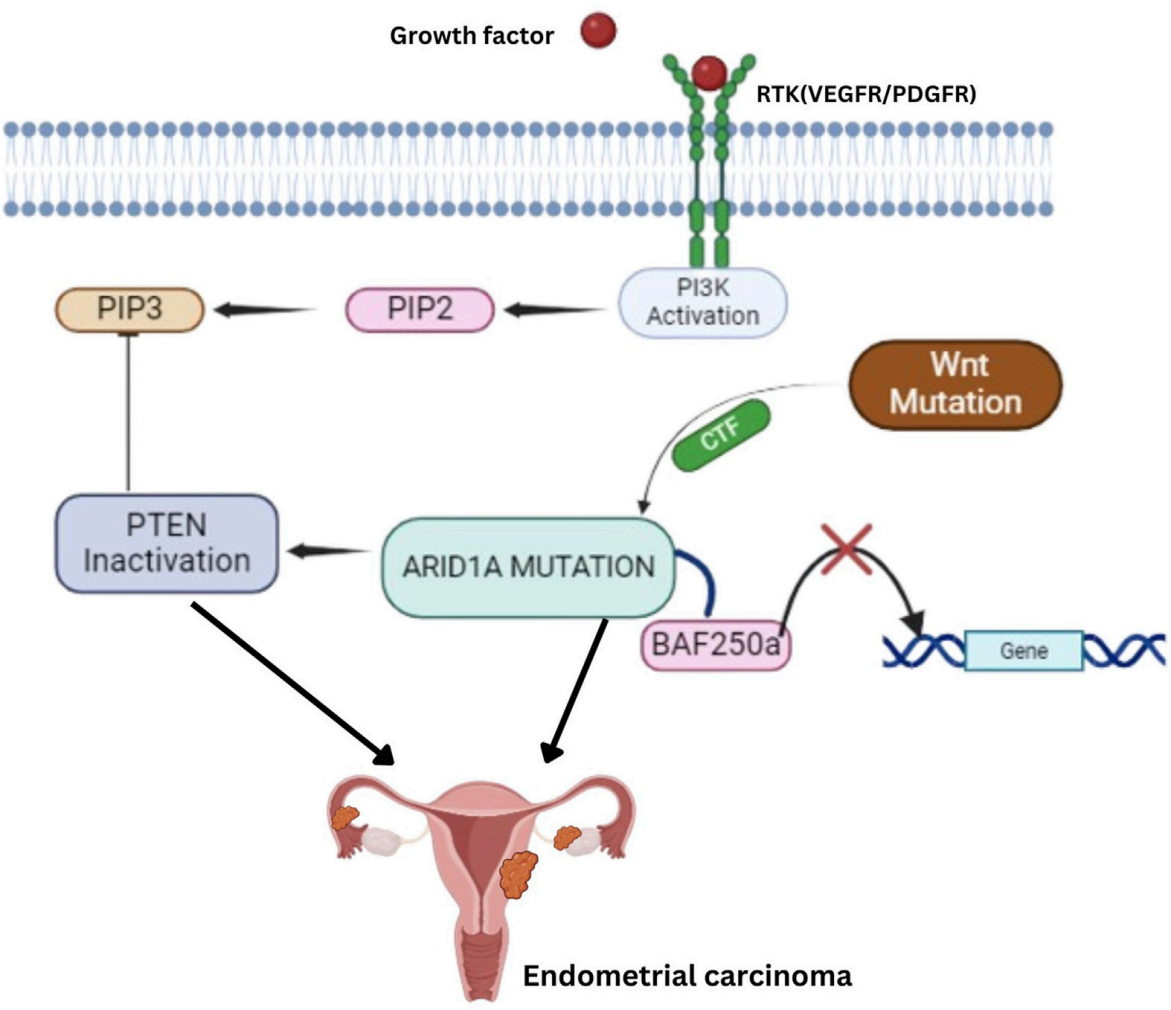
Key Molecular Pathways in Endometrial Carcinoma: ARID1A, PTEN, and Wnt Signaling Mutations, and PI3K Activation lead to Tumorigenesis. Receptor Tyrosine Kinases (RTK) – Growth factors, such as VEGFR/PDGFR, activate RTKs, triggering the PI3K pathway. PI3K Activation–This leads to the conversion of PIP2 to PIP3, which PTEN normally regulates. However, PTEN inactivation disrupts this control, contributing to tumorigenesis. ARID1A Mutation–Mutations in ARID1A disrupt the function of the BAF250a complex, a critical player in chromatin remodeling, further contributing to gene dysregulation and cancer progression. Wnt Pathway Mutation–Mutations in the Wnt signaling pathway also play a role by activating downstream targets that promote cell proliferation and inhibit normal gene regulatory mechanisms.

EECs also feature alterations in the RAS-RAF-MAPK pathway, with *KRAS* mutations present in 18% of cases, often coexisting with mutations in *PTEN, PIK3CA*, and *PIK3R1* (65). *BRAF* mutations are rare, occurring in only 1% of EECs. fibroblast growth factor receptor 2 (FGFR2) mutations, found in 12% of EECs, are mostly missense mutations and are mutually exclusive with *KRAS* mutations but frequently co-occur with *PTEN* mutations, making FGFR2 a potential therapeutic target (66, 67). The WNT signaling pathway is frequently disrupted through *CTNNB1* (β-catenin) mutations in up to 45% of EECs (68). Additionally, *ARID1A* gene mutations, affecting the BAF250a component of the switch/sucrose nonfermenting (SWI/SNF) chromatin-remodeling complex, are found in approximately 40% of low-grade and 39% of high-grade EECs (Figure 3) (69).

Serous endometrial carcinomas (ECs) exhibit distinct genetic profiles and clinical behaviors compared to EECs (70). Serous ECs are often characterized by aneuploidy and frequent alterations such as *TP53* mutations, overexpression of *Cyclin-E* and *Erb-B2* Receptor Tyrosine Kinase 2 (*ERBB2)*, and p16 dysregulation (71, 72). *TP53* mutations are the most common genetic changes in serous ECs, occurring in 53%–90% of tumors, and are often found in early precancerous stages, suggesting a stepwise progression to malignancy (73). These mutations are less common in EECs, with a higher frequency in high-grade cases. The protein phosphatase 2 scaffold subunit Alpha (*PPP2R1A*) gene, which encodes the scaffolding subunit of the protein phosphatase-2A (*PP2A*) enzyme, is also frequently mutated in serous ECs (17%–41%) but less so in EECs (5%–7%). These mutations may impair *PP2A*’s tumor suppressor function, potentially contributing to tumorigenesis (74–76).

The overexpression and amplification of HER-2/ERBB2 are notably more prevalent in serous endometrial carcinomas (ECs) compared to endometrioid endometrial carcinomas (EECs). Research indicates that *HER-2/ERBB2* overexpression occurs in 17%–80% of serous EC cases, with gene amplification reported in 17%–42% of these tumors (77, 78). *HER-2/ERBB2* status in serous ECs is associated with shorter survival times, suggesting its predictive value (79, 80). Additionally, *HER-2/ERBB2*-positive serous ECs are more frequently observed in patients with a previous history of breast cancer (81).

### 7.1 Epigenetic disruption in LS-Associated EC: critical role of aberrant methylation

Aberrant methylation patterns play a critical role in the tumorigenesis of EC, particularly in cases associated with LS. Hypermethylation of tumor suppressor genes and hypomethylation of oncogenes disrupt key cellular pathways, including proliferation, apoptosis, and immune evasion (82). The *MLH1* gene is frequently hypermethylated in EC, especially in MSI-H tumors. This methylation silences *MLH1* expression, impairing the DNA mismatch repair pathway and allowing the accumulation of genetic mutations. This deficiency in mismatch repair is a hallmark of LS-associated EC, resulting in a high mutational burden and tumor heterogeneity (83). Other tumor suppressor genes commonly affected by hypermethylation include *PTEN, RASSF1A*, and *CDKN2A.* Hypermethylation of the *PTEN* promoter reduces its expression, disrupting the PI3K/AKT pathway, which contributes to uncontrolled cellular proliferation and survival (84). Similarly, hypermethylation of *RASSF1A* silences its role in regulating cell cycle arrest and apoptosis, thereby enhancing cell proliferation and suppressing apoptotic signaling. Methylation of *CDKN2A* silences this cyclin-dependent kinase inhibitor, disrupting cell cycle regulation and enabling unchecked cellular growth (84). In contrast, global DNA hypomethylation can activate oncogenes such as *C-MYC*, which promotes increased proliferation, metabolic reprogramming, and evasion of apoptosis. Additionally, hypomethylation of MEST (Mesoderm-Specific Transcript) leads to its overexpression, enhancing oncogenic signaling and tumor progression (85).

In the context of hormone signaling, hypermethylation of *HOXA10* and *HOXA11*, genes essential for endometrial development, disrupts critical pathways involved in maintaining endometrial homeostasis. These changes alter estrogen receptor (ER) and progesterone receptor (PR) signaling, further contributing to hormone-driven progression of EC (86). Methylation also modulates immune response pathways, as seen with the hypermethylation of *SOCS3* (Suppressor of Cytokine Signaling 3), which promotes immune evasion by altering cytokine signalling (87). The clinical implications of these methylation changes in EC are profound. Hypermethylated genes such as *MLH1, PTEN*, and *RASSF1A* show promise as diagnostic biomarkers for early detection of EC. Methylation patterns of genes like *CDKN2A* and *MLH1* also serve as prognostic indicators, correlating with tumor stage, grade, and patient outcomes. Notably, MSI-H EC tumors, characterized by *MLH1* hypermethylation, often respond favorably to immunotherapy due to their high mutational burden and resultant neoantigen expression (88).

## 8 Genetic mutation and pathways alteration driving CRC progression

Ahadova et al. (2018) proposed that three distinct pathways explain CRC development in Lynch patients, in contrast to the widely accepted idea that mutations in the Wnt/β-catenin pathway underlie all CRC development in LS. The three signalling pathways frequently affected in LS CRCs are the Wnt/β-catenin the RAF/MEK/ERK and the PI3K/PTEN/AKT pathways, all of which aid in a cell’s road to malignancy when in a deregulated state (89). *APC* mutations are distributed across the gene and both alleles need to be affected, while *CTNNB1* shows gain-of-function mutations usually located in exon 3, an exon that encodes a regulatory domain normally phosphorylated by *GSK-3B* (90). Additionally, polymorphisms in *CCND1, TP53, IGF1*, and *AURKA* influenced age-associated risk for CRC in LS. Reeves et al 2008. confirmed that the IGF1 polymorphism is an important modifier of disease onset in LS. Talseth et al 2008. reported that the *CCND1* polymorphism was associated with a significant difference in age of disease onset in patients harboring *MSH2* mutations, which was not observed in *MLH1* mutation carriers. A shorter CA-repeats is associated with an earlier age at onset of CRC in LS (91–93). The pathway-based approach of Chen et al. 2009. to elucidate genetic risk modifiers influencing age of onset of CRC in patients with LS using CART analysis (classification and regression tree) identified *CDKN2A C580T* and *IGF1* CA-repeat as the initial splits, indicating that the polymorphisms in these genes are the most informative for separating patients into those LS patients who are more likely to develop CRC early versus those who are more likely to develop CRC at a later age. The gene–gene interaction between *E2F2* and *AURKA* as the influence of the *AURKA* SNP on risk varies depending on the *E2F2* genotype (94). A particularly notable finding is that individuals with biallelic mutations in the *MUTYH* gene face a significantly elevated lifetime risk of developing CRC, with estimates ranging from a 28-fold increase reported by Lubbe et al. (2009) (95). Similarly, to a 93-fold increase was reported and a near-complete penetrance by the age of 60. Moreover, even monoallelic carriers of pathogenic or likely pathogenic *MUTYH* variants exhibit a moderately increased CRC risk—approximately 1.68-fold. Some monoallelic carriers also harbored mutations in other base excision repair (BER) genes, such as *OGG1* and *MTH1*, underscoring the role that alterations in low-penetrance genes may play in CRC development (96, 97). Statistical analyses from the research findings estimate that approximately 15 or fewer of these mutations are critical drivers of tumor development. Key driver genes in CRC include *APC, KRAS, NRAS, BRAF, PIK3CA*, and *PTEN* (98, 99). *APC* acts as a gatekeeper gene, initiating adenoma formation when mutated. Approximately 40% of CRC harbor *KRAS* mutations, predominantly at codons 12 and 13, which are critical in the progression of advanced CRC cells (100). *NRAS* mutations, although less common, occur at codons 12, 13, or 61. *BRAF* mutations, found in 5%–10% of CRCs, are associated with the CpG island methylator phenotype (CIMP) and an altered adenoma-carcinoma progression pathway (101). The interplay between these mutations and the resulting disruptions in signaling pathways provides valuable insights into the mechanisms of CRC development and progression, paving the way for targeted treatments and better diagnostic tools (102).

## 9 Mechanism of cancer initiation in EC and progression to CRC

Although LS is primarily driven by mutations in MMR genes (*MLH1, MSH2, MSH6*, and *PMS2*), several other genes contribute to EC development in LS patients. These genes regulate crucial cellular processes such as tumor suppression, chromatin remodeling, and cell signaling, which, when disrupted, accelerate tumorigenesis. One of the earliest molecular events in LS-associated EC is the inactivation of *PTEN*. Loss of *PTEN* function results in uncontrolled cell proliferation, increased survival, and resistance to apoptosis, hallmark features of cancer progression. Like sporadic EC, *PTEN* mutations are common in LS-associated cases and contribute to early tumorigenesis (103). Additionally, MSI-induced frameshift mutations in *TGFBR2* disrupt *TGF-β* signaling, which normally functions as a tumor suppressor by regulating cell growth and differentiation. The disruption of this pathway allows for uncontrolled cellular proliferation. The loss of *TGF-β* signaling leads to unchecked cell proliferation and enhances tumor progression (104). The PI3K/AKT signaling pathway is further affected by mutations in *PIK3CA. PIK3CA* mutations contribute to sustained activation of the pathway, driving tumor growth and increasing resistance to apoptosis (105). Additionally, *ARID1A*, a chromatin remodeling gene, is frequently mutated in MSI-H tumors, including LS-associated EC. Loss of *ARID1A* function disrupts DNA repair mechanisms, leading to genomic instability and increased tumor mutation rates (106). Other oncogenic mutations found in LS-associated EC include *KRAS*, which affects the RAS/MAPK signaling pathway and promotes uncontrolled cell growth (107, 108). Additionally, overexpression of *SOX9*, a transcription factor involved in stem cell maintenance and differentiation, has been linked to increased tumorigenicity in MSI-H EC (109).

**FIGURE 4:**
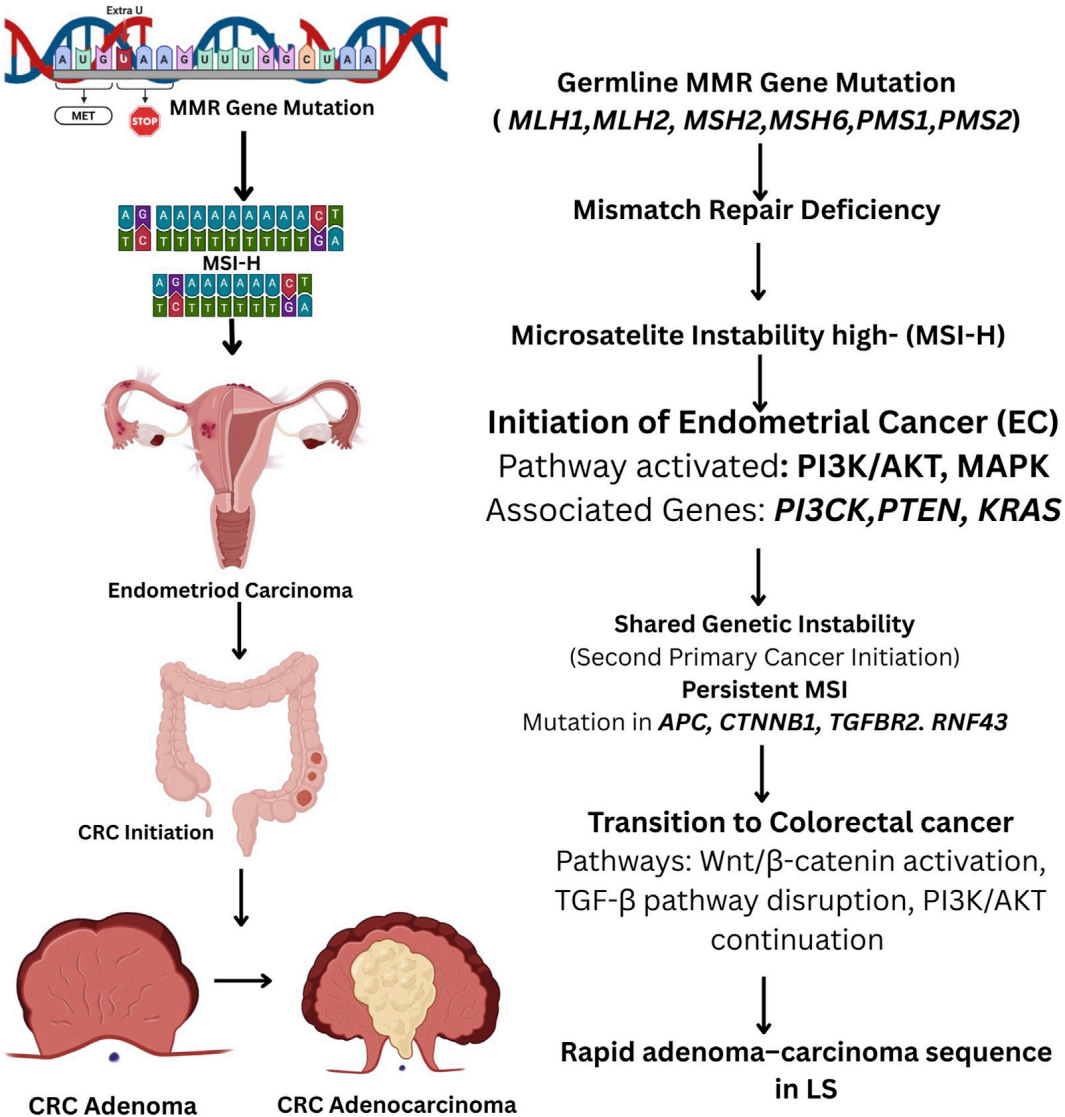
Flowchart illustrating the molecular progression of endometrial cancer (EC) to colorectal cancer (CRC) in Lynch Syndrome (LS). Germline mutations in mismatch repair (MMR) genes (MLH1, MSH2, MSH6, PMS2, EPCAM) lead to MMR deficiency and microsatellite instability (MSI). This instability triggers the initiation of EC via activation of the PI3K/AKT and MAPK pathways, with involvement of genes such as PTEN, PIK3CA, and KRAS. Persistent MSI results in secondary malignancies, including CRC, through mutations in APC, CTNNB1, TGFBR2, and RNF43, promoting Wnt/β-catenin activation, TGF-β pathway disruption, and continued PI3K/AKT signaling. The rapid adenoma–carcinoma sequence in LS accelerates CRC progression.

The Wnt/β-catenin signaling pathway, a critical cell proliferation and differentiation regulator, is frequently altered in LS-associated ECMutations in *CTNNB1*, which encodes *β-catenin*, lead to aberrant activation of this pathway, further supporting tumorigenesis (110). Additionally, *RNF43*, a gene that negatively regulates Wnt signaling, is often mutated in MSI-H ECs, further enhancing tumor growth; the prevalence of truncating mutations at this locus, combined with the rarity of synonymous mutations, strongly indicates that *RNF43* mutations have been positively selected during the evolution of EC and CRC (Figure 4) (111). Given the shared genetic basis of LS-associated EC and CRC, it is likely that their molecular pathways exhibit significant similarities. In approximately 50% of LS cases, EC is diagnosed before CRC in instances where the two malignancies are not synchronous, rendering CRC the second primary cancer in these patients. This sequential pattern of cancer development is likely driven by LS’s shared underlying genetic alterations characteristic. As a result, EC may function as a sentinel malignancy, serving as an early indicator of LS in affected individuals and facilitating the identification of at-risk family members through genetic screening and surveillance (38, 112).

## 10 Uncovering the CRC risk in EC: clinical implications

Following the diagnosis of EC as the primary cancer, individuals may face a heightened risk of developing a second primary cancer due to the shared genetic predispositions, environmental exposures, or the impact of treatments for the initial cancer. To address this, there is an immediate necessity for recommendations based on clinical evidence that focus on preventive strategies, including regular screening for secondary cancers among those who have survived from the primary cancer (113). Individuals with LS face up to an 80% lifetime risk of developing CRC (114). Therefore, genetic testing and CRC screening are strongly recommended, if any family member is diagnosed with LS (115, 116).

In 2012, a study (Singh et al 2012) was conducted to assess CRC in women diagnosed with EC. The research comprised a total of 267 women with EC, of whom 2.4% were found to have CRC. Additionally, 13.6% had significant pathological findings, such as adenomatous polyps and tubulovillous histology (117). After that Singh et al. (2013) performed a study that included 3,115 women with EC found that women under 50 years of age had a significantly higher risk of developing CRC of any type, with a hazard ratio (HR) of 4.41 and a 95% confidence interval (CI). The risk was particularly elevated for right-sided CRC, with an HR of 7.48 and a 95% CI. In contrast, no elevated risk of CRC was noted in women aged 51–65 years or older than 65 years. However, women aged 51–65 years with EC had an increased risk of right-sided CRC, with an HR of 2.30 and a 95% CI (116). Another study by Win et al. (2013) reported that women with EC carrying mutations in MMR genes had an elevated risk of developing CRC within the next 20 years. The estimated probability of CRC development was 48%, with a 95% CI. The study also identified a significantly increased risk of CRC, as indicated by a standardized incidence ratio (SIR) of 39.9 (95% CI) compared to the normal population (118). A retrospective cohort study by Liao SC et al. (2021) found that the prevalence of CRC in women with EC was 2.20 times higher compared to controls, with an incidence rate of 1.09 per 1000 person–years. The study also noted that the risk of CRC increased with age, and the hazard ratio for CRC development was highest within 3 years of an EC diagnosis (119). Further (Lai et al., 2021), women diagnosed with EC exhibited significantly elevated SIRs for CRC, irrespective of age. In a sub-site-specific analysis of CRC, EC patients diagnosed before the age of 50 demonstrated higher SIRs for ascending colon. The cumulative incidence of second primary malignancies in EC patients was evaluated over 5, 10, 15, and 20 years of follow-up. Notably, the incidence of CRC showed a progressive increase, rising from 0.7% at 5 years to 3.9% at 20 years. Patients aged ≥50 consistently exhibited a higher incidence than those aged <50, with rates reaching 5.7% and 2.1%, respectively, at 20 years. These findings suggest that EC survivors, particularly those aged ≥50, are at an increased long-term risk of developing CRC (120). This highlights the critical need for ongoing surveillance, risk assessment, and the implementation of targeted preventive strategies in this high-risk population.

## 11 Prognostic markers of EC and CRC

Predictive biomarkers are crucial in predicting disease progression, independent of treatment. These markers are measurable clinical or biological characteristics that provide insight into a patient’s likely outcome. In EC, blood-based prognostic biomarkers have garnered significant interest among healthcare professionals and patients due to their potential for easy assessment (121). Two protein-based biomarkers have emerged as particularly noteworthy in EC prognosis: Human Epididymis protein 4 (HE4) and cancer antigen 125 (CA125). CA125, in particular, has been the focus of multiple investigations. An increasing amount of evidence indicates a correlation between elevated serum CA125 levels and unfavorable clinicopathological features in EC patients (122). Furthermore, research indicates that higher CA125 concentrations may be associated with poorer outcomes in individuals diagnosed with EC (123). HE4 is a glycoprotein that was initially identified in the epididymis but has been shown to be highly expressed in various cancer types, including EC (Table 1) (124).

**TABLE 1:** Biomarkers in endometrial cancer: types, descriptions, and clinical advantages.

Marker	Type	Description	Advantage	Reference
Endometrial cancer				
CA125	Protein	Serum biomarker; elevated levels correlate with unfavorable clinicopathological features	Easy blood-based measurement for patient monitoring	(130)
HE4	Protein	Glycoprotein overexpressed in several cancer types, including EC	Higher specificity compared to CA125 for EC progression	(131)
Hormone Receptor Status (ER/PR)	Protein	Estrogen and progesterone receptor positivity	Indicator for favorable prognosis and less aggressive treatment	(131)
P^53^	Genetic	Mutations in tumor suppressor gene p53	Correlates with poor clinical outcomes; Strong predictor of aggressive cancer behavior and high risk	(131, 133)
HER2	Genetic	Gene amplification, more common in serous histology	Identifies patients who may benefit from HER2-targeted therapies	(132)
MSI	Genetic	DNA mismatch repair deficiency	Potential marker for immunotherapy response	(133)
PI3K/AKT/m TOR pathway	Genetic	Affected in over 80% of type I ECs; includes PTEN and PIK3CA mutations	Presents both prognostic significance and therapeutic potential	(132)

Hormone receptor status, particularly progesterone receptor (PR) and estrogen (ER) positivity, has been recognized as a key prognostic marker linked to a substantial enhancement in disease-free survival. Mutations in the tumor suppressor gene *p53* are prominent in type II ECs, with studies reporting mutations in up to 90% of serous carcinomas. *p53* mutations correlate with poor clinical outcomes, including an 11-fold elevated risk of death in multivariate analyses adjusting for lymph node metastasis, grade, histology, and FIGO stage (125). *HER2* gene amplification, more common in serous histology, has been identified as a distinct prognostic marker associated with reduced overall survival. The PI3K/AKT/mTOR pathway, affected in over 80% of type I ECs, presents both prognostic significance and therapeutic potential, with *PTEN* and *PIK3CA* mutations being key components (126). MSI has shown conflicting prognostic implications, with some studies reporting improved 5-year survival rates, while other studies observed no notable variation in relapse or overall survival. These molecular markers and emerging factors, such as microvascular proliferation, are refining our ability to predict EC outcomes and guide personalized treatment strategies (127).

At this point, CRC patient prognosis depends on clinicopathological parameters, with an emphasis on the cancer stage upon diagnosis. The total 5-year rate of survival for stage I is above 90%; it decreases to 70% for the second stage, 58% for stage III, and fewer than 15% for stage IV (128). Prognostic markers are crucial in predicting outcomes and guiding treatment decisions for CRC patients. Carcinoembryonic antigen (CEA), despite its limitations in specificity and accuracy, has shown potential as a distinct prognostic marker for all stages of CRC. A large-scale National Cancer Database data study suggested that serum CEA serves as a reliable prognostic indicator for stage II tumor recurrence (129). Epidermal growth factor receptor (*EGFR*) expression is observed in 50%–70% of CRC, although its prognostic significance remains inconclusive (130). The homeobox protein *CDX2* has emerged as a promising marker of colon cancer cell differentiation and a robust prognostic indicator (Table 2) (131).

**TABLE 2:** Biomarkers in colorectal cancer: types, descriptions, and clinical advantages.

Marker	Type	Description	Advantage	Reference
Colorectal Cancer				
CEA	Protein	Serum biomarker. Independent prognostic factor, useful for recurrence detection in stage II CRC.	Widely used and non-invasive, useful for tracking recurrence	(134)
EGFR	Protein	Expressed in 50%–70% of CRCs	Potential therapeutic target in CRC, guiding treatment	(135)
CDX2	Protein	Homeobox protein. Marker of colon cancer differentiation and prognosis	Indicator of better differentiation and prognosis in CRC.	(134)
Ki67	Protien	Cell proliferation marker	Correlates with tumor proliferation, useful in predicting disease aggressiveness	(136)
KRAS	Genetic	Tyrosine kinase downstream of EGFR, Validated as a predictive biomarker for treatment decisions	Predicts response to EGFR-targeted therapies	(137, 138)

Interestingly, conflicting evidence exists regarding *Ki67* expression in CRCs, with some studies suggesting that high expression is associated with good clinical outcomes. At the same time, a meta-analysis showed a strong association between elevated *Ki-6*7 expression and reduced overall survival. disease-free survival (132). *KRAS*, a tyrosine kinase downstream of the EGFR receptor, stands out as the first validated predictive biomarker in colon cancer. These various prognostic markers collectively enhance our understanding of CRC mechanisms and assist in customizing treatment strategies to achieve better patient outcomes (2, 133).

## 12 Targeted therapeutics for EC in Lynch syndrome

In the treatment of EC, especially among patients with LS, therapeutic approaches are customized based on the cancer’s progression and the associated risk of relapse. Radiotherapy is often employed in early-stage EC, while chemotherapy is indicated for cases with high-grade histology or advanced chronic conditions (134). As the disease progresses, the risk of recurrence increases significantly. Notably, recurrent vaginal EC tends to respond well to treatments, frequently utilizing radiation therapy as an effective option. Surgery remains the cornerstone of EC treatment, with total hysterectomy (TH) and bilateral salpingo-oophorectomy (BSO) being the standard procedures (135). TH involves the removal of the uterus and cervix, while BSO eliminates the fallopian tubes and ovaries. In patients with LS, oophorectomy is routinely performed during surgery to exclude the presence of ovarian metastases or primary ovarian tumors, given the elevated risk associated with LS (134). Current surgical options include open surgery, laparotomy, and minimally invasive techniques such as laparoscopic surgery (LS) and robot-assisted surgery (RS), which have demonstrated efficacy and reduced recovery times. For advanced stages, particularly Stage III EC, chemotherapy regimens typically include paclitaxel and carboplatin, with alternatives like ifosfamide combined with paclitaxel or cisplatin being explored (136).

Emerging therapeutic pathways in preclinical studies focus on targeting specific molecular mechanisms involved in EC progression, such as cell cycle inhibition, *EZH2* inhibition, and modulation of the prorenin pathway. These innovative strategies aim to enhance treatment efficacy and are increasingly integral to personalized medicine approaches. Recent advancements in immunotherapy have notably transformed the treatment landscape for advanced EC (137). The U.S. Food and Drug Administration (FDA) has approved several immune checkpoint inhibitors for this indication, marking significant progress in therapy options for patients, particularly those with dMMR tumors. Notable approvals include durvalumab (Imfinzi) for patients with mismatch repair-deficient tumors, pembrolizumab (Keytruda) for use irrespective of dMMR status, and dostarlimab (Jemperli), which has also been approved for dMMR advanced EC (135). These agents can be employed as first-line therapies or for recurrent cancer after specific prior treatments, significantly expanding the arsenal of options for managing advanced EC and improving patient outcomes, especially for those who previously had limited immunotherapy choices.

## 13 Targeted therapeutics for CRC in Lynch syndrome

Surgical resection continues to be the foremost intervention for CRC, especially in individuals diagnosed with LS, who exhibit an elevated propensity for the onset of CRC at earlier ages and frequently present with more advanced stages of the disease. In scenarios where the malignancy is classified as non-resectable, a multimodal approach encompassing chemotherapy, radiation therapy, and immunotherapy is generally utilized (139, 140). Radiation therapy constitutes an essential element of CRC management, particularly in the context of rectal cancer, as it employs high-energy X-rays to selectively target and obliterate neoplastic cells through the induction of DNA damage, thereby impeding cellular growth and proliferation (141, 142). This modality proves especially advantageous for rectal tumors that are confined, rendering them more susceptible to radiation intervention. Contemporary chemotherapy protocols for CRC frequently incorporate fluoropyrimidine-based agents, such as 5-fluorouracil (5-FU), in conjunction with combination therapies involving agents such as oxaliplatin (OX), irinotecan (IRI), and capecitabine (143).

Recent innovations in the management of advanced CRC have increasingly centered on targeted therapies, particularly those aimed at inhibiting angiogenesis. Monoclonal antibodies, including bevacizumab, ramucirumab, and aflibercept, have demonstrated a capacity to improve overall survival metrics when administered alongside standard chemotherapy regimens, offering substantial advantages for patients afflicted with advanced disease, including those with LS (144). Oral therapeutic agents such as regorafenib and trifluridine/tipiracil have surfaced as viable alternatives for patients exhibiting refractory CRC, presenting renewed optimism for individuals with constrained treatment options. Although the survival enhancements associated with these novel agents may appear to be modest, they signify considerable progress within the therapeutic domain, enabling patients to potentially experience extended survival and enhanced quality of life (145). Furthermore, research has indicated a plausible role for estrogen/progestin replacement therapy in postmenopausal women, with antecedent findings suggesting a reduced incidence of CRC linked to these therapies, albeit the underlying mechanisms remain elusive. Given the intersection of hormonal influences and cancer risk, further investigation into this association may be warranted, particularly in LS patients who encounter augmented risks for various malignancies, including CRC (77, 146). Overall, the ongoing advancement of therapeutic strategies for CRC, particularly within the framework of LS, underscores the necessity of personalized treatment modalities customized to the distinct genetic and molecular attributes of tumors, thereby enhancing patient outcomes and survival probabilities.

## 14 Summarizing the landscape of Lynch syndrome-associated cancers

This review underscores the critical link between EC and subsequent CRC in individuals with LS, primarily driven by mutations in MMR genes and the presence of MSI. By consolidating findings from the original research data, it highlights the importance of early identification, genetic screening, and vigilant surveillance in high-risk populations. Both cancers are influenced by common genetic pathways, including the Wnt and PI3K/AKT/mTOR signaling cascades, with critical mutations in genes such as *APC, PTEN*, and *β-catenin*. The progression and development of these malignancies are also significantly impacted by lifestyle factors like obesity and hormonal imbalances. Understanding the molecular and genetic commonalities between EC and CRC is crucial for early diagnosis and the formulation of personalized treatment strategies. For patients with LS, the heightened risk of both cancers underscores the need for genetic counselling and regular screenings for these tumors. Advances in diagnostic techniques, including molecular biomarkers and high-throughput omics technologies, have enhanced the detection, treatment, and prognosis of both cancers. Furthermore, emerging therapeutic approaches, especially in the realm of targeted therapy and immunotherapy presents promising opportunities for better patient results. The connection between EC and CRC underscores the need for a comprehensive approach to managing patients, particularly those with genetic predispositions, to mitigate risks and enhance survival rates. This comprehensive review may provide a reference for clinicians and researchers aiming to refine diagnostic and management approaches in LS and explore future advancements in oncology.

## 15 Future directions in treating EC and CRC in LS

Exploring genetic and molecular interconnections between EC and CRC, especially in LS, is crucial for advancing research and treatment. Identifying shared signaling pathways will facilitate the creation of effective targeted therapies tailored for LS patients. Incorporating these findings into clinical practice may enhance affected individuals’ survival rates and quality of life. Immunotherapy, particularly checkpoint inhibitors, is a promising research area for treating EC and CRC in patients with LS and mismatch repair deficiencies. Exploring immune modulation and combination therapies could lead to innovative strategies that enhance immune responses against these cancers, improving outcomes for advanced-stage patients. Advances in next-generation sequencing (NGS) and high-throughput omics technologies will aid in the discovery of new biomarkers for early diagnosis and prognosis.

## Glossary

LS: Lynch Syndrome
EC: Endometrial cancer
CRC: Colorectal Cancer
MMR: Mismatch Repair
MSS: Microsatelite Stable
MSI: Microsatelite Instability
MSI-H: Microsatelite Instability-High
MSI-L: Microsatelite Instability-Low
MLH1: MutL homolog 1
MLH2: MutL homolog 2
MSH2: MutS homolog 2
MSH6: MutS homolog 6
PMS1: PMS1 homolog 1
PMS2: PMS1 homolog 2
EPCAM: Epithelial Cell Adhesion Molecule
PTEN: Phosphatase and Tensin Homolog
TGF-βR2: Transforming growth factor, beta receptor II
BAX: BCL-2-associated X protein
HR: Hazard Ratio
PD-1: Programmed Death −1
PD-L1: Programmed Death Ligand 1
KRAS: Kirsten rat sarcoma viral oncogene homologue
PIK3CA: phosphatidylinositol-4,5-bisphosphate 3-kinase catalytic subunit alpha
BMI: Body Mass Index
APC: Adenomatous polyposis coli
CTNNB1: Catenin beta-1
PCOS: Polycystic ovary syndrome
HRT: Hormone Replacement Therapy
IBD: Inflammatory Bowel Disease
TCGA: The Cancer Genome Atlas
DEGs: Differentially Expressed Genes
LS-CRC: Lynch Syndrome Associated Colorectal Cancer
LS-EC: Lynch Syndrome Associated Endometrial Cancer
COL11A1: Collagen Type XI Alpha 1 Chain
SG-LC: Specific Genes overlaps LS and CRC
SG-LE: Specific Genes overlaps LS and EC
CST2: Type 2 cystatin
COL18A1: Collagen Type XVIII Alpha 1 Chain
LY6K: Lymphocyte Antigen 6 Family Member K
MIR27B: MicroRNA 27b
SST: Somatostatin
KIF20A: Kinesin-like protein
NUF2: Kinetochore protein Nuf2
HTR4: 5-Hydroxytryptamine receptor 4
CDC45: Cell Division Cycle 45
WDR31: WD Repeat Domain 31
AADACL2: Arylacetamide Deacetylase Like 2
DHRS7C: Dehydrogenase/Reductase 7C
KRT24: keratin gene
LINC00460: Long Intergenic Non-Protein Coding RNA 460
NPY2R: Neuropeptide Y receptor type 2
KHDRBS2: KH RNA Binding Domain Containing, Signal Transduction Associated 2
CDH10: Cadherin 10
LINC02616: Long Intergenic Non-Protein Coding RNA 2616
LINC02691: Long Intergenic Non-Protein Coding RNA 2691
IGF2-AS: IGF2 Antisense RNA
IGF: Insulin-like growth factor
ADAMTS9-AS2: ADAM Metallopeptidase With Thrombospondin Type 1 Motif 9- Antisense RNA 2
SLC10A4: Solute Carrier Family 10 Member 4
EECs: Endometrioid Endometrial Carcinomas
BHD: Birt–Hogg–Dubé syndrome
IGFIIR: Insulin-like Growth Factor II Receptor
Rad3: A yeast homolog of human ATR
ATR: Ataxia Telangiectasia and Rad3-related protein
*PIK3R1*: Phosphoinositide-3-Kinase Regulatory Subunit 1
BRAF: B-Raf Proto-Oncogene, Serine/Threonine Kinase
FGFR2: Fibroblast Growth Factor Receptor 2
ARID1A: AT-Rich Interaction Domain 1A
BAF250a: BRG1-Associated Factor 250a
SWI/SNF: SWItch/Sucrose Non-Fermentable
ERBB2: Erb-B2 Receptor Tyrosine Kinase 2
PPP2R1A: Protein Phosphatase 2 Scaffold Subunit A Alpha
PP2A: Protein Phosphatase 2A
HER-2: Human Epidermal Growth Factor Receptor 2
RASSF1A: Ras Association Domain Family Member 1 isoform A
CDKN2A: Cyclin Dependent Kinase Inhibitor 2A
C-MYC: MYC Proto-Oncogene, BHLH Transcription Factor
MEST: Mesoderm Specific Transcript
HOXA10: Homeobox A10
HOXA11: Homeobox A11
SOCS3: Suppressor of Cytokine Signaling 3
GSK-3B: Glycogen Synthase Kinase 3 Beta
CCND1: Cyclin D1
SIR: Standardized Incidence Rate
CI: Confidence Interval
TP53: Tumor Protein P53
AURKA: Aurora Kinase A
CART: Cocaine- and Amphetamine-Regulated Transcript
E2F2: E2F Transcription Factor 2 (involved in cell cycle regulation)
SNP: Single Nucleotide Polymorphism
MUTYH: MutY DNA Glycosylase
BER: Base Excision Repair
OGG1: 8-Oxoguanine DNA Glycosylase
CIMP: CpG Island Methylator Phenotype
HE4: Human Epididymis Protein 4
CA125: Cancer Antigen 125
PR: Progesterone Receptor
ER: Estrogen Receptor
FIGO: International Federation of Gynecology and Obstetrics
CDX2: Caudal Type Homeobox 2
Ki67: Marker of Cellular Proliferation
TH: Total Hysterectomy
LS: Laproscopic Surgery
RS: Robot-assisted Surgery
BSO: Bilateral Salpingo-Oophorectomy
RS: Recurrence Score
EZH2: Enhancer of Zeste Homolog 2
FDA: Food and Drug Administration
dMMR: Deficient Mismatch Repair
5-FU: 5-Fluorouracil
OX: Oxaliplatin
IRI: Irinotecan
NGS: Next-Generation Sequencing
